# Supplementary material for: The Evaluation of Significance of Uncoupling Protein Genes UCP1, UCP2, UCP3, UCP4, UCP5, and UCP6 in Human Adaptation to Cold Climates
Source: Biology (Basel). 2025 Apr 23;14(5):454. doi: 10.3390/biology14050454 (PMC12108989; doi:10.3390/biology14050454)
Supplement: Supplementary file 1 [file biology-14-00454-s001.zip › biology-3548192-supplementary.pdf]

Supplementary Material

The evaluation of significance of uncoupling protein genes UCP1, UCP2, UCP3, UCP4, UCP5 and UCP6 in human adaptation to cold climates

TABLE OF CONTENTS

Chapter S1	2
Research design	2
Chapter S2	3
Genotype and allele frequencies of 9 polymorphic variants of uncoupling protein *UCPs* genes	3
Chapter S3	4
Associative analysis of polymorphic variants of *UCP* genes with hormone levels of the pituitary-thyroid axis (TSH, FT3 and FT4)	4
Chapter S4	7
Associative analysis of polymorphic variants of *UCP* genes with changes in thyroid homeostasis (SPINA)	7
Chapter S5	8
Associative analysis of polymorphic variants of *UCP* genes with BSA	8
Chapter S6	9
Distribution of the natural selection signals for polymorphic variants of *UCP* genes	9

Chapter S1

Research design

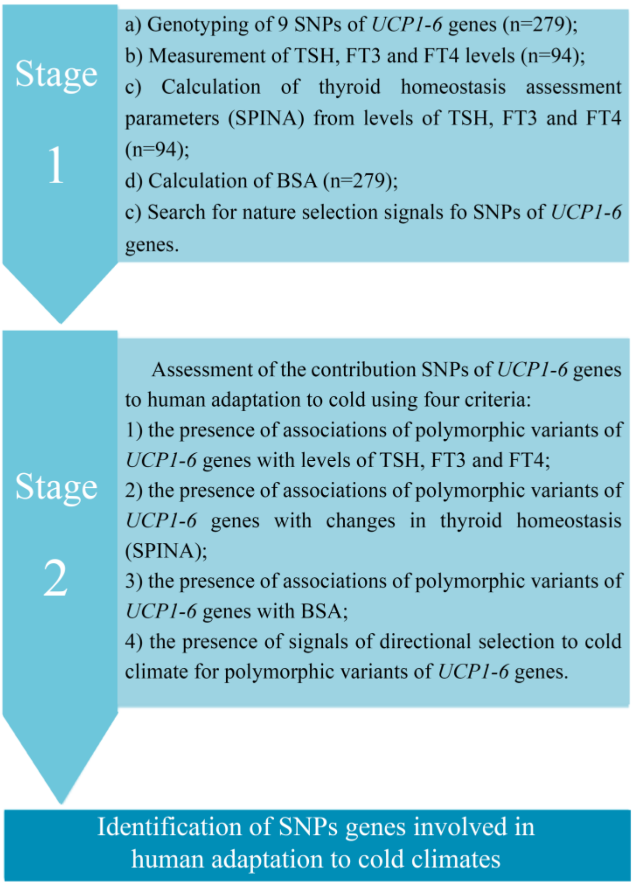

Figure S1. Study design

Chapter S2

Genotype and allele frequencies of 9 polymorphic variants of uncoupling protein UCP genes

Genotype and allele frequencies of the 9 polymorphic variants of the *UCP1-6* genes are summarized in Table S1. The distribution of genotype frequencies of 9 polymorphic variants of *UCP1-6* genes in the sample consisted of 279 individuals to the Hardy-Weinberg equilibrium (Table S1).

Table S1. Genotype and allele frequencies of 9 polymorphic variants of *UCP1-6* genes in the Yakut population and their conformity to the Hardy-Weinberg equilibrium (HWE).

№	SNP	Gene	Genotype, n			Allele frequencies		HWE[1]	
								χ^2	p
1	rs1800592	*UCP1*	AA<br>109	AG<br>126	GG<br>44	A<br>0.616	G<br>0.384	0.563	0.453
2	rs3811787		TT<br>101	TG<br>132	GG<br>46	T<br>0.60	G<br>0.40	0.070	0.796
3	rs659366	*UCP2*	TT<br>74	TC<br>134	CC<br>71	T<br>0.505	C<br>0.495	0.431	0.511
4	rs660339		AA<br>75	AG<br>123	GG<br>81	A<br>0.49	G<br>0.51	3.876	0.049
5	rs1800849	*UCP3*	TT<br>85	TC<br>127	CC<br>67	T<br>0.53	C<br>0.47	2.054	0.152
6	rs2075577		AA<br>124	AG<br>120	GG<br>35	A<br>0.66	G<br>0.34	0.500	0.479
7	rs9472817	*UCP4*	CC<br>80	CG<br>128	GG<br>71	C<br>0.52	G<br>0.48	1.852	0.174
8	rs1010978	*UCP5*[2]	T<br>63		C<br>31	T<br>0.67	C<br>0.33	94	3.156
9	rs9526067	*UCP6*	AA<br>184	AT<br>86	TT<br>9	A<br>0.81	T<br>0.19	0.457	0.499

Note: [1] Hardy-Weinberg distribution (HWE); [2] The analysis of the frequencies of genotypes and alleles was carried out for male (n=94).

Chapter S3

Associative analysis of polymorphic variants of UCP genes with hormone levels of the pituitary-thyroid axis (TSH, FT3 and FT4)

To reveal the relationship of 9 polymorphic variants of *UCP1-6* genes with the levels of hormones of pituitary-thyroid axis (TSH, FT3 and FT4) the total sample of individuals (n = 94) was aligned by the median of each hormone (TSH 2.01 μU/mL; FT3 6.52 pmol/L; FT4 14.6 pmol/L) to normalize the sample due to thyroid allostasis. For each hormone, the sample was divided into two groups, Group I included individuals with values less than the median (TSH < 2.01 μU/mL (n = 39); FT3 < 6.52 pmol/L (n = 42); FT4 < 14.6 pmol/L (n = 40)) and Group II included individuals with values above the median (TSH > 2.01 μU/mL (n = 38); FT3 > 6.52 pmol/L (n = 35); FT4 >14.6 pmol/L (n = 38)).

As a result of the analysis in individuals from group I, the associations for polymorphism rs1800849 of *UCP3* gene with FT3 levels were found, where carriers of TT genotype had significantly higher FT3 levels (6.1 ± 0.29 pmol/L; p = 0.05) compared to the opposite CC genotype (5.69 ± 0.34 pmol/L) (Table S2).

Table S2. Association analysis of 9 polymorphic variants of *UCP1-6* genes with the levels of pituitary-thyroid hormones TSH, FT3 and FT4 in subjects from the group I.

**Gene, SNP**	**Genotypes, mean ± st.dev.**			**H; *p***
UCP1, rs1800592	GG	AG	AA	
TSH	1.58±0.47	1.51±0.41	1.59±0.36	H=0.3005336; *p* = 0.8605
FT3	5.71±0.25	5.92±0.35	5.97±0.48	H =2.022399; *p* = 0.3638
FT4	13.18±1.24	13.1±1.25	13.01±1.20	H=0.0842454; *p* =0.9588
UCP1, rs3811787	TT	GT	GG	
TSH	1.55±0.37	1.52±0.44	1.7±0.34	H =0.8217832; *p* =0.663
FT3	5.96±0.44	5.88±0.37	5.91±0.44	H=0.6162211; *p* =0.7348
FT4	13.13±0.99	13.09±1.41	12.94±1.25	H=0.2824113; *p* =0.8683
UCP2, rs659366	CC	CT	TT	
TSH	1.69±0.34	1.52±0.41	1.51±0.4	H =1.157988; *p* =0.5605
FT3	5.86±0.43	5.96±0.39	5.89±0.40	H=0.5095306; *p* =0.7751
FT4	13.13±1.10	12.73±1.14	13.40±1.32	H=2.947061; *p* =0.2291
UCP2, rs660339	AA	AG	GG	
TSH	1.55±0.40	1.52±0.41	1.66±0.35	H =0.6351490; *p* =0.7279
FT3	5.89±0.40	5.97±0.39	5.82±0.44	H =1.053086; *p* =0.5906
FT4	13.29±1.33	12.86±1.08	13.06±1.19	H=1.492140; *p* =0.4742
UCP3, rs1800849	TT	TC	CC	
TSH	1.42±0.41	1.64±0.35	1.51±0.44	H=2.215982; *p* =0.3302
FT3	6.10±0.29	5.91±0.43	5.69±0.34	H =5.963700; *p* =0.05
FT4	13.38±1.15	13.01±1.41	12.86±0.83	H=1.335577; *p* =0.5128
UCP3, rs2075577	GG	GA	AA	
TSH	1.47±0.43	1.60±0.40	1.52±0.39	H =0.3010469; *p* =0.8603
FT3	5.68±0.62	5.88±0.38	5.99±0.37	H=1.494956; *p* =0.4736
FT4	12.68±0.86	13.11±1.11	13.15±1.38	H=1.012515; *p* =0.6027

UCP4, rs9472817	GG	GC	CC	
TSH	1.66±0.38	1.54±0.37	1.47±0.45	H =0.8740791; *p* = 0.6459
FT3	5.82±0.36	5.96±0.41	5.90±0.42	H=0.9829789; *p* = 0.6117
FT4	13.22±1.13	12.98±1.32	13.15±1.11	H=0.0810359; *p* = 0.9603
UCP5, rs1010978*	C	T		
TSH	1.55±0.43	1.55±0.37		U =171.50; *p* = 0.9184
FT3	5.98±0.36	5.88±0.41		U=152.00; *p* = 0.4356
FT4	13.33±1.11	12.93±1.25		U =156.00; *p* = 0.3788
UCP6, rs9526067	AA	AT	TT	
TSH	1.53±0.41	1.63±0.32	1.75	H =0.4289438; *p* = 0.8070
FT3	5.90±0.43	5.92±0.27	6.2	H=0.7543408; *p* = 0.6858
FT4	12.97±1.31	13.41±0.85	13.2	H=0.5926596; *p* = 0.7435

Note: * - Comparative analysis between two genotypes and alleles was carried out using the ann-Whitney U test

In group II, there were associations for rs3811787 of the *UCP1* gene, rs1800849 of *UCP3* gene and rs1010978 of the *UCP5* gene with FT3 levels and rs9526067 of the *UCP6* gene with TSH levels (Table S3). For the rs3811787 polymorphism of the *UCP1* gene, carriers of the homozygous TT genotype (7.26 ± 0.30 pmol/L; p = 0.02) had statistically significantly higher FT3 levels than carriers of the heterozygous GT genotype (6.98 ± 0.35 pmol/L) and homozygous GG genotype (6.85 ± 0.33 pmol/L). For the rs1800849 polymorphism of the *UCP3* gene, carriers of the homozygous TT genotype(6.1 ± 0.29 pmol/L; p = 0.05) had statistically significantly higher FT3 levels than carriers of the heterozygous CC genotype (5.69 ± 0.34 pmol/L). Carriers of the T allele (7.22 ± 0.34 pmol/L; p = 0.02) of rs1010978 of the *UCP5* gene had significantly higher levels of FT3 than carriers of the C allele (6.92 ± 0.31 pmol/L). For rs9526067 of the *UCP6* gene, a significant increase in TSH levels was found in AA genotype carriers (2.63 ± 0.53 μU/mL; p = 0.02) compared to TT+TA genotype (3.22 ± 0.53 μU/mL).

Table S3. Association analysis of 9 polymorphic variants of *UCP1-6* genes with levels of pituitary-thyroid hormones TSH, FT3 and FT4 in subjects from the group II.

Gene, SNP	Genotypes, mean±st.dev.			H; *p*
UCP1, rs1800592	GG	AG	AA	
TSH	2.89±0.73	2.84±0.65	2.90±0.67	H=0.0664712; *p* =0.9673
FT3	5.17±0.25	5.92±0.35	5.97±0.48	H=2.022399; *p* =0.3638
FT4	15.25±0.07	15.73±0.53	15.53±0.59	H=2.719948; *p* =0.2567
UCP1, rs3811787	TT	GT	GG	
TSH	2.95±0.72	2.88±0.62	2.75±0.70	H=0.4313535; *p* =0.8060
FT3	7.26±0.30	6.98±0.35	6.85±0.33	H=7.871803; *p* =0.02
FT4	15.58±0.57	15.66±0.54	15.53±0.65	H=0.4597523; *p* =0.7946
UCP2, rs659366	CC	CT	TT	
TSH	2.78±0.72	2.68±0.62	3.15±0.59	H=4.646130; *p* =0.0980
FT3	5.86±0.43	5.96±0.39	5.89±0.40	H=0.5095306; *p* =0.7751
FT4	15.53±0.59	15.6±0.48	15.68±0.69	H=0.2724607; *p* =0.8726
UCP2, rs660339	AA	AG	GG	
TSH	3.09±0.62	2.86±0.68	2.59±0.63	H=4.136481; *p* =0.1264

FT3	5.89±0.40	5.98±0.39	5.82±0.44	H=1.053086; *p* =0.5906
FT4	15.68±0.69	15.59±0.49	15.55±0.55	H=.2065173; *p* =0.9019
UCP3, rs1800849	TT	TC	CC	
TSH	2.92±0.59	2.96±0.65	2.72±0.77	H=1.658699; *p* =0.4363
FT3	6.1±0.29	5.91±0.43	5.69±0.34	H=5.963700; *p* =0.05
FT4	15.96±0.61	15.52±0.53	15.39±0.37	H=3.559775; *p* =0.1687
UCP3, rs2075577	GG	GA	AA	
TSH	2.43±0.72	2.98±0.70	2.91±0.56	H=4.217837; *p* =0.1214
FT3	5.68±0.62	5.88±0.38	5.99±0.37	H=1.494956; *p* =0.4736
FT4	15.30±0.33	15.46±0.50	15.89±0.57	H=5.636911; *p* =0.07
UCP4, rs9472817	GG	GC	CC	
TSH	2.54±0.62	2.98±0.68	3.11±0.56	H=4.804767; *p* =0.0905
FT3	5.83±0.36	5.96±0.41	5.90±0.42	H=0.9829789; *p* =0.6117
FT4	15.59±0.54	15.52±0.58	15.84±0.50	H =2.30938; *p* =0.3152
UCP5, rs1010978*	C	T		
TSH	3.07±0.73	2.79±0.62		U=116.00; *p* =0.2091
FT3	6.92±0.31	7.22±0.34		U=74.50; *p* =0.02
FT4	15.39±0.44	15.70±0.58		U=92.00; *p* =0.09
UCP6, rs9526067	AA	AT	TT	
TSH	2.63±0.53	3.17±0.67	3.93	H=7.997978; *p* =0.02
FT3	5.91±0.42	5.92±0.27	6.2	H=0.7543408; *p* =0.6858
FT4	15.59±0.52	15.64±0.64	15.6	H=0.0356739; *p* =0.9823

Note: * - Comparative analysis between two alleles was carried out using the Mann-Whitney U test

Chapter S4

Associative analysis of polymorphic variants of UCP genes with changes in thyroid homeostasis (SPINA)

As a result of SPINA parameter calculations, normal values of SPINA-GT parameter were found in all examined individuals (2.63 ± 0.74 pmol/s). For the SPINA-GD parameter 56 (65%) individuals were found to have elevated values of SPINA-GD (45.70 ± 3.95 nmol/s), while the remaining 30 (35%) individuals had values of this parameter within the normal range (36.45 ± 2.79 nmol/s). An associative analysis of 9 polymorphic variants of *UCP1-6* genes with SPINA parameters was performed in male (n = 61), taking into account elevated levels of FT3 and FT4, seasonal characteristics (excluded persons who donated blood in the spring) and body mass index (excluded persons with underweight and overweight). Significant associations of polymorphic variant *UCP6* (rs9526067) with SPINA-GT, and polymorphisms *UCP1* (rs3811787) with SPINA-GD were found.

Table S4. Associative analysis of genotypes of 9 polymorphic variants of *UCP1-6* genes with SPINA parameters

Gene, SNP	Genotypes, mean±st.dev.			H; *p*
UCP1, rs1800592	GG	AG	AA	
SPINA-GT	2.53±0.79	2.77±0.79	2.51±0.73	H = 2.2699; *p* = 0.3214
SPINA-GD	44.45±6.29	41.30±6.50	43.38±4.50	H = 3.4311; *p* = 0.1799
UCP1, rs3811787	TT	GT	GG	
SPINA-GT	2.60±0.75	2.67±0.80	2.44±0.71	H = 0.9724; *p* = 0.6150
SPINA-GD	44.56±5.12	40.43±5.71	44.74±6.45	H = 7.9872; *p* = 0.02
UCP2, rs659366	CC	CT	TT	
SPINA-GT	2.55±0.54	2.71±0.84	2.49±0.80	H=1.006391 *p* =0.6046
SPINA-GD	43.81±5.03	43.52±6.41	40.93±5.37	H =2.216743 *p* =0.3301
UCP2, rs660339	AA	AG	GG	
SPINA-GT	2.47±0.76	2.70±0.86	2.63±0.51	H = 1.8994; *p* = 0.3869
SPINA-GD	42.00±6.49	42.86±5.74	43.93±5.24	H = 1.3119; *p* = 0.5189
UCP3, rs1800849	TT	TC	CC	
SPINA-GT	2.56±0.77	2.61±0.70	2.64±0.89	H = 0.2892; *p* = 0.8654
SPINA-GD	44.30±7.26	42.51±4.33	41.76±6.67	H = 1.2145; *p* = 0.5448
UCP3, rs2075577	GG	GA	AA	
SPINA-GT	2.64±0.63	2.60±0.79	2.60±0.79	H = 0.1207; *p* = 0.9414
SPINA-GD	43.99±6.43	41.54±4.80	44.09±6.71	H = 2.9474; *p* = 0.2291
UCP4, rs9472817	GG	GC	CC	
SPINA-GT	2.65±0.61	2.55±0.75	2.66±0.96	H = 1.2206; *p* = 0.5432
SPINA-GD	43.14±4.76	43.48±6.52	41.03±5.48	H = 1.6446; *p* = 0.4394
UCP5, rs1010978*	C	T		
SPINA-GT	2.58±0.68	2.62±0.80		U = 415.00; *p* = 0.9395
SPINA-GD	42.99±5.20	42.74±6.21		U = 415.00; *p* =0.9395
UCP6, rs9526067*	AA	AT±TT		
SPINA-GT	2.78±0.82	2.28±0.51		U= 259.00; *p* = 0.01
SPINA-GD	42.77±5.78	42.93±6.09		U = 409.00; *p* = 0.8674

Note: * - Comparative analysis between two genotypes and alleles was carried out using the Mann-Whitney U test

Chapter S5

Associative analysis of polymorphic variants of UCP genes with BSA

The distribution of genotypes and allele frequencies of 9 polymorphic variants of *UCP1-6* genes between body surface areas (BSA) were calculated for 279 individuals (Table S5). We found associations of the rs1808049 genotypes of the *UCP3* gene with BSA.

Table S5. Associative analysis of 9 polymorphic variants of *UCP1-6* genes with BSA

Gene, SNP	Genotypes, mean±st.dev.			H; *p*
UCP1, rs1800592<br>BSA, м²	GG<br>1.69±0.21	AG<br>1.63±0.18	AA<br>1.65±0.17	H = 3.3014; *p* = 0.1919
UCP1, rs3811787<br>BSA, м²	TT<br>1.66±0.18	GT<br>1.64±0.19	GG<br>1.64±0.17	H = 1.2495; *p* = 0.5354
UCP2, rs659366<br>BSA, м²	CC<br>1.64±0.15	CT<br>1.62±0.18	TT<br>1.68±0.20	H = 4.6680; *p* = 0.0969
UCP2, rs660339<br>BSA, м²	AA<br>1.69±0.20	AG<br>1.63±0.19	GG<br>1.63±0.16	H =4 .8819; *p* = 0.0871
UCP3, rs1800849<br>BSA, м²	TT<br>1.61±0.17	TC<br>1.64±0.18	CC<br>1.69±0.20	H = 6.6479; *p* = 0.0360
UCP3, rs2075577<br>BSA, м²	GG<br>1.64±0.15	GA<br>1.66±0.19	AA<br>1.63±0.19	H = 2.8499; *p* = 0.2405
UCP4, rs9472817<br>BSA, м²	GG<br>1.63±0.19	GC<br>1.67±0.19	CC<br>1.62±0.15	H = 2.0419; *p* = 0.3603
UCP5, rs1010978*<br>BSA, м²	C<br>1.81±0.15	T<br>1.77±0.18	-	U = 759.50; *p* = 0.0809
UCP6, rs9526067<br>BSA, м²	AA<br>1.64±0.18	AT<br>1.64±0.18	TT<br>1.66±0.25	H = 0.0286; *p* = 0.9858

Note: * - Comparative analysis between two alleles was carried out using the Mann-Whitney U test and 94 male.

Chapter S6

Distribution of the natural selection signals for polymorphic variants of UCP genes

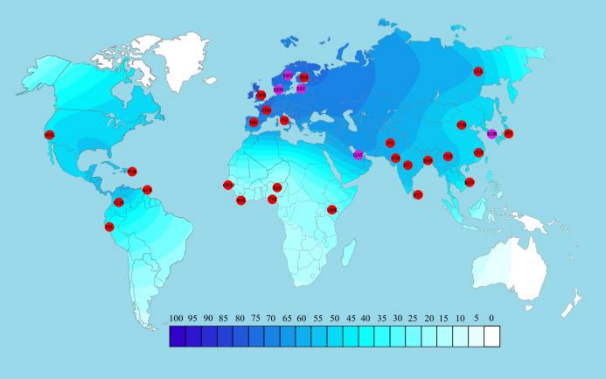

A-allele rs1800592 gene *UCP1*

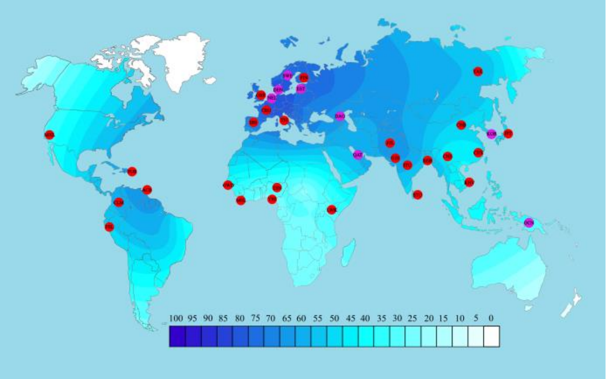

T-allele rs3811787 gene *UCP1*

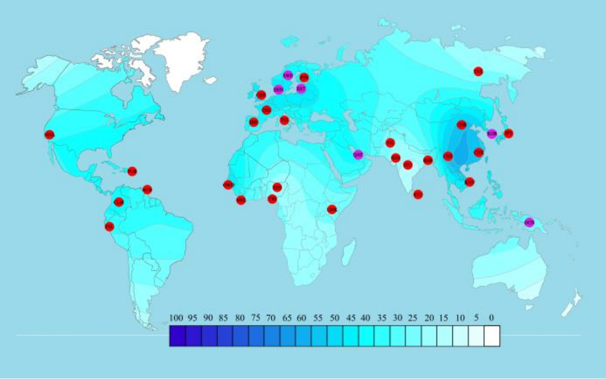

T-allele rs659366 gene *UCP2*

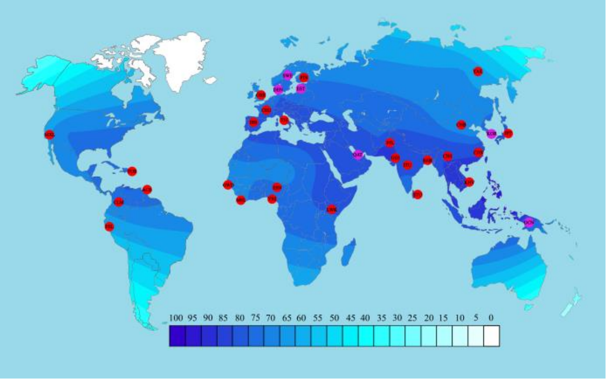

G-allele rs660339 gene *UCP2*

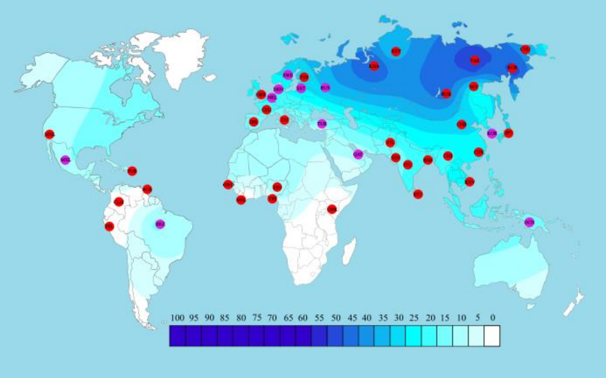

T-allele rs1800849 gene *UCP3*

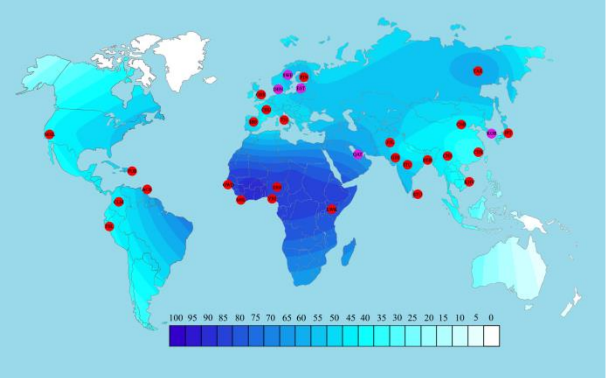

A-allele rs2075577 gene *UCP3*

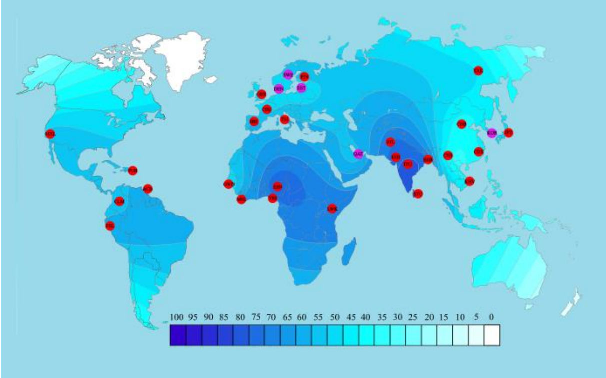

C-allele rs9472817 gene *UCP4*

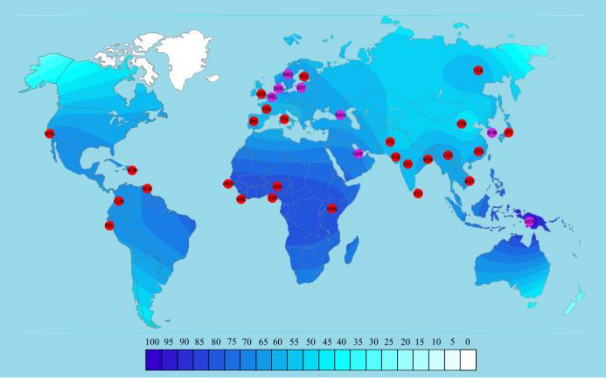

T-allele rs1010978 gene *UCP5*

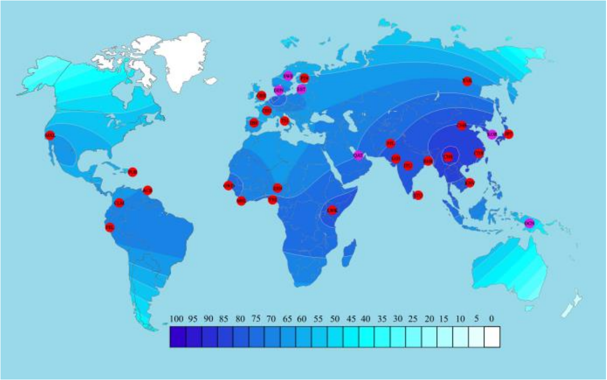

A-allele rs9526067 gene *UCP6*

Figure S2. Distribution of allele frequencies of 9 polymorphic variants of *UCP1-6* genes.
